# Evaluation of reference genes for real-time quantitative PCR studies in *Candida glabrata* following azole treatment

**DOI:** 10.1186/1471-2199-13-22

**Published:** 2012-06-29

**Authors:** Qingdi Quentin Li, Jeff Skinner, John E Bennett

**Affiliations:** 1 Clinical Mycology Section, Laboratory of Clinical Infectious Diseases, National Institute of Allergy and Infectious Diseases, National Institutes of Health, Bethesda, MD, 20892, USA; 2 Bioinformatics and Computational Biosciences Branch, Office of Cyber Infrastructure and Computational Biology, National Institute of Allergy and Infectious Diseases, National Institutes of Health, Bethesda, MD, 20892, USA

**Keywords:** Candida glabrata, Azole resistance gene, Fluconazole, hkgFinder, Housekeeping gene, Reference gene, RT-qPCR

## Abstract

**Background:**

The selection of stable and suitable reference genes for real-time quantitative PCR (RT-qPCR) is a crucial prerequisite for reliable gene expression analysis under different experimental conditions. The present study aimed to identify reference genes as internal controls for gene expression studies by RT-qPCR in azole-stimulated *Candida glabrata*.

**Results:**

The expression stability of 16 reference genes under fluconazole stress was evaluated using fold change and standard deviation computations with the hkgFinder tool. Our data revealed that the mRNA expression levels of three ribosomal RNAs (*RDN5.8*, *RDN18*, and *RDN25*) remained stable in response to fluconazole, while PGK1, UBC7, and UBC13 mRNAs showed only approximately 2.9-, 3.0-, and 2.5-fold induction by azole, respectively. By contrast, mRNA levels of the other 10 reference genes (*ACT1*, *EF1α*, *GAPDH*, *PPIA*, *RPL2A*, *RPL10*, *RPL13A*, *SDHA*, *TUB1*, and *UBC4*) were dramatically increased in *C. glabrata* following antifungal treatment, exhibiting changes ranging from 4.5- to 32.7-fold. We also assessed the expression stability of these reference genes using the 2^-ΔΔCT^ method and three other software packages. The stability rankings of the reference genes by geNorm and the 2^-ΔΔCT^ method were identical to those by hkgFinder, whereas the stability rankings by BestKeeper and NormFinder were notably different. We then validated the suitability of six candidate reference genes (*ACT1*, *PGK1*, *RDN5.8*, *RDN18*, *UBC7*, and *UBC13*) as internal controls for ten target genes in this system using the comparative C_T_ method. Our validation experiments passed for all six reference genes analyzed except *RDN18*, where the amplification efficiency of *RDN18* was different from that of the ten target genes. Finally, we demonstrated that the relative quantification of target gene expression varied according to the endogenous control used, highlighting the importance of the choice of internal controls in such experiments.

**Conclusions:**

We recommend the use of *RDN5.8*, *UBC13*, and *PGK1* alone or the combination of *RDN5.8* plus *UBC13* or *PGK1* as reference genes for RT-qPCR analysis of gene expression in *C. glabrata* following azole treatment. In contrast, we show that *ACT1* and other commonly used reference genes (*GAPDH*, *PPIA*, *RPL13A*, *TUB1*, etc.) were not validated as good internal controls in the current model.

## Background

The investigation of gene expression has become increasingly prevalent in numerous animal, human, microorganism, and plant studies [1-5]. The quantitation of gene expression requires sensitive, precise, and reproducible measurements for specific mRNA sequences. Generally, gene expression levels can be determined by a variety of techniques, including Northern blotting, RNase protection assay, semi-quantitative reverse-transcription PCR, and real-time quantitative PCR (RT-qPCR) [4]. RT-qPCR has gained favor as it is a highly sensitive, accurate, and fast technique that offers high-throughput and the ability to detect low-abundance mRNAs [6] and quantify mRNA copy number [7]. Thus, RT-qPCR has been used for countless different applications [1-5].

One of the main uses of RT-qPCR, when coupled with reverse transcription, is to measure gene expression at the mRNA level in various biological samples. However, there is substantial technical variability associated with RT-qPCR, arising from inherent differences in samples, sample collection, RNA degradation and extraction efficiency, quantity and quality of input RNA, reverse transcription and PCR efficiency, and pipetting accuracy or error. Researchers have employed a number of strategies to normalize their data, including normalization to (*i*) genomic DNA, (*ii*) total RNA, (*iii*) an external standard, and (*iv*) a reference gene. The most common practice is to normalize to an internal control gene termed a reference gene. A reference gene is subject to the same errors in cDNA preparation as the gene of interest, making it an excellent normalizing control. However, selection of an inappropriate reference gene can add large unpredictable error to the analysis and result in incorrect estimates [8]. The ideal reference gene should have a stable RNA transcription level under different experimental conditions and be sufficiently abundant across different tissues and cell types. However, it has become apparent that such an ideal reference gene has not yet been identified [9]. The most commonly used reference genes, including β-actin, cyclophilin, GAPDH, tubulin, and 18S and 28S ribosomal RNAs, have shown variable expression levels in different cells and tissues under different conditions, and therefore they are unsuitable for normalization purposes owing to large measurement error [6,10-33]. Hence, it is no longer acceptable to arbitrarily select any reference gene for normalization; it must be demonstrated that the reference gene of choice is suitable for the experiment in question.

In recent decades, *Candida glabrata* has emerged as the second most common cause of invasive fungal infection [1,34,35]. Azoles such as fluconazole are the first-line drugs for the treatment of fungal infections caused by *C. glabrata*. However, resistance to azoles can arise rapidly in *C. glabrata* during treatment of patients with azoles [36]. An increasing body of evidence has implicated ATP-binding cassette transporters (e.g., Cdr1 and Pdr1) and sterol biosynthetic enzymes (e.g., Erg3 and Erg11) in azole resistance in *C. glabrata* in both clinical and laboratory settings [1,34-37]. The expression of these genes in *C. glabrata* in response to azoles is not completely understood. Therefore, we set out to establish an in vitro model for investigating azole-inducible gene expression in *C. glabrata*, using RT-qPCR. For reliable gene expression analysis, a compulsory step is the selection of good reference genes for normalization; however, no validated reference genes have been reported for the relative quantification of the mRNA expression profile in *C. glabrata* following exposure to azoles.

We have been using *ACT1* as the internal control for gene expression analysis by RT-qPCR in clinical isolates of *C. glabrata* in the absence of drug challenges [1]. Other researchers also use *ACT1* as the reference gene for azole-inducible gene expression studies by slot blotting in *Candida* species [34,37,38]. However, the suitability of *ACT1* in studies of azole-inducible gene expression in *C. glabrata* has not been validated. In this work, we evaluated 16 reference genes to establish their suitability as control genes for normalization and identified a set of genes that are suitable for quantitative gene expression analysis by RT-qPCR in *C. glabrata* following fluconazole treatment.

## Methods

### Cell culture and drug treatment

All five *C. glabrata* strains (Table 1) used in the present study were grown in YPD broth (Difco Laboratories, Detroit, MI, USA) at 30°C with shaking at 225 rpm. The *ura3* mutant Cg84u did not grow in minimal (MIN) medium, unless supplemented with 20 μg/ml of uracil (Sigma-Aldrich, St. Louis, MO, USA).

**Table 1:** ***Candida glabrata*** **strains used in this study**

**Strain**	**Parental strain**	**Genotype or description**	**Fluconazole MIC (μg/mL)**	**Reference**
**NCCLS84**		Wild-type (ATCC90030)*	64	[39]
**Cg84u**	NCCLS84	Δ*ura3*	256	[40]
**CgB4**	Cg84u	*Δura3* Δ*Cgpdr1*::Tn5 <; Cm	1	[35]
**Cg3S**		Clinical susceptible isolate	32	[36]
**Cg4R**		Clinical resistant isolate	>256	[36]

*American Type Culture Collection, Manassas, VA, USA.

Fluconazole (Euroasian Chemicals Private Ltd., Fort, Mumbai, India) was added to cultures of each strain at a final concentration of 200 μg/ml, followed by continued incubation with shaking for 2 h. Cell cultures without fluconazole treatment served as controls.

The susceptibility of each *C. glabrata* strain to fluconazole was determined on YPD agar medium using an E-test (AB Biodisk, Solna, Sweden) according to the manufacturer’s instructions (Table 1).

### RNA isolation and reverse transcription

Total RNA was extracted from *C. glabrata* logarithmic-phase cultures grown in YPD broth, using TRIzol reagent (Invitrogen, Life Technologies, Grand Island, NY, USA) according to the manufacturer’s instructions. The concentration and purity of the RNA was determined using a UV spectrophotometer (NanoDrop 2000C; ThermoFisher Scientific, Waltham, MA, USA) by measuring the absorbance at 230 (OD_230_), 260 (OD_260_) and 280 nm (OD_280_). The OD_260nm_/OD_280nm_ of the samples, reflecting the average purity, ranged from 1.80 to 2.05, and the OD_260nm_/OD_230nm_ was in the range of 2.00–2.60. The integrity of the RNA was further checked in a selected subset of samples by electrophoresis through 1% denaturing and non-denaturing agarose gels.

Reverse transcription (RT) was performed on 1 μg of total RNA using a commercially available kit. Prior to RT, the total RNA samples were treated with DNase for 30 min at 37°C (TURBO DNA-free; Ambion, Life Technologies, Grand Island, NY, USA) according to the manufacturer’s instructions. RNA was converted to cDNA using the High Capacity cDNA Reverse Transcription Kit (Applied Biosystems, Life Technologies, Carlsbad, CA, USA). The reaction took place in a thermal cycler (T3 Thermocycler; Biometra, Goettingen, Germany) with a single cycle and incubation periods of 25°C for 10 min, 37°C for 120 min, and 85°C for 5 min. All investigated samples were transcribed with the same reverse transcription reaction conditions. Negative controls, which were run simultaneously, did not contain either RNA (no template control) or no reverse transcriptase (RT negative control), to control for RNA and genomic DNA contamination, respectively.

### Primer and probe design

Primers and probes were designed in our laboratory using the primer analysis software Primer Express 3.0 (Applied Biosystems). TaqMan probes were synthesized by Applied Biosystems, and primers were synthesized by Invitrogen/Life Technologies. The primers and TaqMan probes used in the current study were selected to bind specifically to the cDNAs of Cg84u and other *C. glabrata* strains (Table 1). The sequences of TaqMan probes and forward and reverse primers, the gene numbers, and the localization for each PCR assay for the 16 reference genes and 10 target genes assessed in this study are listed in Additional file 1, Additional file 2, and Additional file 3.

### RT-qPCR analysis

RT-qPCR for reference gene RNA transcription was performed by SYBR Green chemistry (SYBR Green PCR Master Mix; Applied Biosystems). The increase in fluorescence of the SYBR Green dye was monitored using a 7500 Real-time PCR System (Applied Biosystems). This technique has been successfully used to validate reference gene expression levels in yeast and other cell types [41]. Primers were used at 300 nM each for specific forward and reverse primers and cDNA at 25 ng in 25-μl reactions. Primer sets for the reference genes (Additional file 2) were used to amplify the open reading frame (ORF) region of the genes according to the following conditions: one cycle of 50°C × 2 min, 95°C × 10 min; followed by 40 cycles of 95°C × 15 s, 60°C × 1 min; with dissociation (a melting curve) during the last cycle of 95°C × 15 s, 60°C × 1 min, 95°C × 15 s. The dissociation protocol to determine the melting curve from 60°C to 95°C for each PCR product was added after thermocycling to verify that each primer pair produced only a single product. All samples gave only a single peak, indicating a single pure product and no primer/dimer formation. Real-time PCR efficiencies were acquired by amplification of a standardized dilution series of the template cDNA and were determined for each gene as the slope of a linear regression model. PCR efficiency was determined by measuring the C_T_ to a specific threshold for a serial dilution of cDNA. The corresponding real-time PCR efficiencies were then calculated according to the equation: $\text{E}=\left(10^{-1/\text{slope}}-1\right)\times 100$. All PCRs displayed efficiencies between 94% and 119%.

To study target gene expression, the amplification was detected in real time using TaqMan chemistry (TaqMan Universal PCR Master Mix; Applied Biosystems) according to the manufacturer’s instructions. RT-qPCR was performed in 96-well microtiter plates with a final volume of 25 μl, using a 7500 Real-time PCR System (Applied Biosystems). Primers were used at 300 nM each for specific forward and reverse primers; probes, at 200 nM; and cDNA, at 25 ng in 25-μl reactions. Primer sets and TaqMan probes for the target genes (Additional file 3) were used to amplify the ORF region of the genes under the following conditions: one cycle of 50°C × 2 min, 95°C × 10 min; and then 40 cycles of 95°C × 15 s, 60°C × 1 min. The parallel amplification between the reference genes and the target genes was confirmed for each with probe-primer sets. To minimize technical (run-to-run) variation between the samples, all samples were analyzed in the same run for both target genes and reference genes.

### Evaluation of reference gene expression stability using four different software packages

Non-normalized gene expression levels from our experimental data were analyzed to evaluate the expression stability of potential reference genes, using four different software programs: hkgFinder [42], geNorm [9,43], BestKeeper [44], and NormFinder [26]. The hkgFinder software computes the pooled standard deviation (SD) of non-normalized expression data from both phenotypes (i.e., azole-treated and untreated *C. glabrata* cells), the fold change (FC) values between the two phenotypes, and Student’s *t*-tests of the log_2_ fold-change values with Holm-adjusted *P*-values. The reference genes with the smallest SD and the smallest, non-significant FC are identified as the best potential reference genes (Additional file 4). The geNorm software computes a stability value (*M*) and a pairwise variation (*V*), which are used to evaluate each individual reference gene candidate or each combination of reference genes, called a normalization factor (NF). The pairwise variability *V* of two genes *j* and *k* is the standard deviation of all log_2_ ratios of *a*_*j*_/*a*_*k*_, while the stability value *M* of gene *j* is the mean of all possible pairwise variations *V*_*jk*_. Graphs of the *M* values help identify the best individual reference genes, and graphs of the *V* values identify the optimal number of reference genes for an NF. Note that an earlier version called geNorm Excel was produced as an add-in for MS Excel and it required several hand calculations to convert crossing point (CP) values into relative expression values. That version is now unavailable and the new geNorm PLUS from Biogazelle does not require those hand calculations. The BestKeeper software uses pairwise correlation to determine whether potential reference genes should be included in a BestKeeper Index, which is simply the geometric mean of the CP or cycle threshold (C_T_) values. The NormFinder software computes a different type of stability value (*ρ*_*ig*_) based on the intragroup and intergroup variation of the expression data. The software instructions from each package were followed when inputting the RT-qPCR data, fetching the output, and interpreting the analysis results.

## Results

### Stability of RNA transcription of reference genes in *C. glabrata* following azole stimulation

In the present study, 16 reference genes were chosen from among commonly used reference genes in published studies with yeast and mammalian cells, paying close attention to selecting genes that belong to different functional classes; their full names, symbols, functions, and gene numbers are listed in Additional file 1. Our aim was to identify reference genes with minimal variability under our experimental conditions. To this end, RT-qPCR was used to measure the RNA transcription levels of 16 reference genes in *C. glabrata* cells following fluconazole treatment. To compare the different RNA transcription levels after azole exposure, the C_T_ values of the reference genes were directly compared between the drug-treated (t) and untreated (ut) samples using the formula: C_T_ Change = C_T_(ut) − C_T_(t). The C_T_ is defined as the number of cycles needed for the fluorescence signal to reach a specific threshold level of detection and is inversely correlated with the amount of template cDNA present in the reaction. Thus, a higher value of C_T_ Change indicates lower stability of a reference gene, considering that the expression of a reference gene should not change significantly with azole treatment. As expected, the RNA transcription levels of the reference genes varied (Table 2). The three ribosomal RNA subunits *RDN5.8*, *RDN18*, and *RDN25* were the most stable reference genes, with C_T_ Change values less than 0.5, while *UBC13*, *PGK1*, and *UBC7* were relatively stable with C_T_ Change values of only around 1.5. By contrast, the other 10 reference genes showed marked variation in response to fluconazole. Among them, the most prominent variation was found in the RNA transcription levels of *SDHA*, *ACT1*, and *RPL13A*; as seen in Table 2, the C_T_ Change values of these reference genes were as high as 5.03.

**Table 2:** **Stability of RNA transcription of reference genes in fluconazole-treated** ***C. glabrata*** **as determined by the 2**^**-ΔΔCT**^**method**

	***RDN18***	***RDN25***	***RDN5.8***	***UBC13***	***PGK1***	***UBC7***	***GAPDH***	***UBC4***	***TUB1***	***EF1α***	***PPIA***	***RPL2A***	***RPL10***	***RPL13A***	***ACT1***	***SDHA***
**C**_**T**_**Change**	0.34	0.39	0.46	1.34	1.56	1.60	2.17	2.41	2.60	2.89	3.13	3.67	4.12	4.17	4.45	5.03
**ΔΔC**_**T**_**(t)**	0.12	0.07	0	-0.87	-1.10	-1.13	-1.71	-1.95	-2.13	-2.43	-2.66	-3.21	-3.65	-3.71	-3.98	-4.57
**2**^**-ΔΔCT**^**(t)**	0.92^#^	0.95^#^	1.00	1.83*	2.14*	2.19*	3.28*	3.86*	4.39*	5.38*	6.32*	9.23*	12.58*	13.06*	15.82*	23.75*
**Ranking**	1	2	3	4	5	6	7	8	9	10	11	12	13	14	15	16

C_T_ Change = C_T_(ut) − C_T_(t); ut, untreated; t, fluconazole-treated.

ΔC_T_ = C_T__reference_ − C_T__RDN5.8_; ΔC_T_ of each reference gene was calculated using *RDN5.8* as the internal control.

ΔΔC_T_(t) = ΔC_T_(t) − ΔC_T_(ut).

2^-ΔΔCT^(t) for fluconazole-treated cells indicates fold change in RNA transcription of a reference gene normalized to *RDN5.8*, as compared with untreated cells.

The stability ranking is based on the values of C_T_ Change, ΔΔC_T_(t), and 2^-ΔΔCT^(t) of the reference genes in fluconazole-treated *C. glabrata* cells.

^#^*P* > 0.05 and *P <; 0.05 for the azole-treated group vs. the untreated group after normalizing to *RDN5.8.*

To validate the stability of candidate RNA transcription under our experimental conditions, the levels were compared with the *RDN5.8* RNA transcription level. We chose to use *RDN5.8* as a normalizer because it meets the requirement for both stability and suitability as a reference. First, we calculated the ΔC_T_ between the C_T_ values of reference genes and *RDN5.8* from fluconazole-treated (t) and untreated (ut) cells:

ΔC_T_(t) = C_T_(t reference) − C_T_(t *RDN5.8*) and ΔC_T_(ut) = C_T_(ut reference) − C_T_(ut *RDN5.8*).

In the second step, we subtracted the change in RNA transcription in untreated samples from the change in treated samples to obtain the ΔΔC_T_(t):

ΔΔC_T_(t) = ΔC_T_(t) − ΔC_T_(ut).

Thus, ΔΔC_T_(t) indicates the change in RNA transcription caused by fluconazole treatment after normalization to RNA transcription changes in *RDN5.8*. A high ΔΔC_T_(t) value indicates a significant fluconazole-related change in the RNA transcription level of the tested gene. A positive ΔΔC_T_(t) value indicates down-regulation of transcription, whereas a negative ΔΔC_T_(t) indicates up-regulation of a gene’s transcription following azole treatment. We then transformed ΔΔC_T_(t) into a 2^-ΔΔCT^ value, which indicates the fold change in RNA transcription of a reference gene in response to fluconazole as compared with the level in untreated cells. The calculated ΔΔC_T_(t) and 2^-ΔΔCT^ values of the 16 tested reference genes in drug-treated samples are given in Table 2.

Following stimulation with fluconazole, the RNA transcription of *SDHA*, *ACT1*, and *RPL13A* was highly regulated in *C. glabrata* cells, with changes ranging from 13- to 23-fold compared with transcription in the untreated cells. There was almost no regulation of *RDN5.8*, *RDN18*, and *RDN25* RNA transcription, while *PGK1*, *UBC7*, and *UBC13* RNA transcription were only approximately 2-fold induction in response to drug treatment (Table 2).

### Determination of reference gene expression stability by four different specific software packages

To choose the best reference genes, the reference gene stability was evaluated using four different software packages: hkgFinder, geNorm, BestKeeper, and NormFinder. Each of these software packages uses a slightly different metric to evaluate the candidate reference genes. Our goal was to compare the findings from these four different methods and look for the best-scoring reference genes that might be common to these different methods.

The hkgFinder software identifies the best reference genes by ranking the candidate genes according to their SD and FC values (Table 3). Among the 16 potential reference genes, the SDs ranged from 0.19 to 2.76, and the FCs ranged from 1.2 to 32.7. The best three reference gene candidates were *RDN18*, *RDN25*, and *RDN5.8*. The next three best candidate reference genes, which also had reasonable SD and FC values, were *UBC13*, *PGK1* and *UBC7*.

**Table 3:** **Assessment of reference gene expression stability in fluconazole-treated** ***C. glabrata*** **by using hkgFinder**

	***RDN18***	***RDN25***	***RDN5.8***	***UBC13***	***PGK1***	***UBC7***	***GAPDH***	***UBC4***	***TUB1***	***EF1α***	***PPIA***	***RPL2A***	***RPL10***	***RPL13A***	***ACT1***	***SDHA***
**SD**	0.19	0.22	0.26	0.73	0.87	0.88	1.19	1.33	1.42	1.58	1.71	2.01	2.26	2.28	2.44	2.76
**Log fold change**	-0.35	-0.39	-0.46	-1.33	-1.56	-1.60	-2.18	-2.41	-2.60	-2.89	-3.12	-3.67	-4.12	-4.17	-4.45	-5.03
**Fold change**	1.2	1.3	1.4	2.5	2.9	3.0	4.5	5.3	6.0	7.4	8.7	12.7	17.4	18.0	21.8	32.7
**Ranking**	1	2	3	4	5	6	7	8	9	10	11	12	13	14	15	16

The fold change shown in the table represents the difference in reference gene expression between azole-treated and untreated *C. glabrata* without normalization to an internal control gene.

The stability ranking is based on the values of standard deviation (SD), log fold change, and fold change of reference genes in fluconazole-treated *C. glabrata* cells. The best reference genes will have the smallest SD and smallest fold-change values.

**Figure 1:**
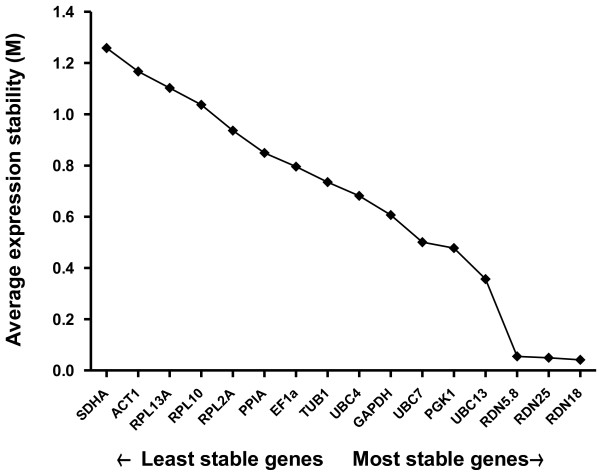
**Determination of the average expression stability (*M*) of the reference genes using the geNorm program.** The reference genes were serially excluded from the analysis, with *M* representing the mean pairwise variation between an individual reference gene and all other tested reference genes. The reference gene indicated at each point on the *x*-axis is the one that is to be excluded from the following step. The most stable reference genes are those that are still included, i.e., those that exhibit the lowest *M* values. Shown is the stability ranking of the reference genes in all samples. Genes are ranked from left to right in order of increasing expression stability (decreasing *M* value)

**Figure 2:**
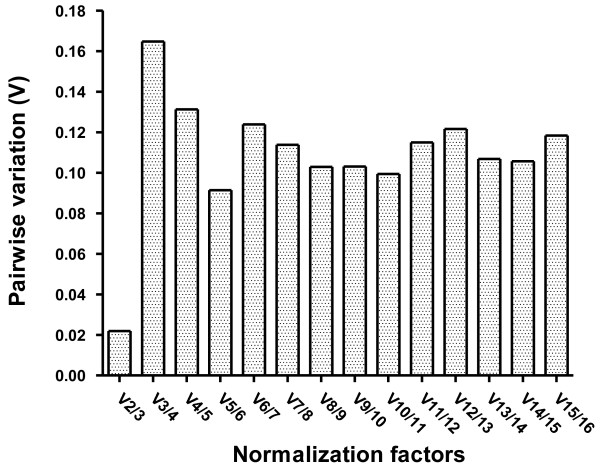
**Determination of the optimal number of reference genes as internal references for normalization using geNorm analysis.** The geNorm program calculates a normalization factor from at least two reference genes and the mean pairwise variation (*V*) between every combination of sequential normalization factors in order to determine the minimum number of reference genes required for accurate normalization in the samples. For example, *V*5/6 represents the comparison of the normalization factors from five and six reference genes, respectively. On the left-most side is the pairwise variation when the number of reference genes is increased from two to three (*V*2/3). Stepwise inclusion of less stable genes generates the subsequent data points. A decrease in the *V* value indicates a positive effect and means that the added gene should preferably be included for calculation of a reliable normalization factor. The cutoff value for *V*, below which the inclusion of an additional reference gene does not result in a significant improvement of normalization, was set at 0.15. It was apparent from the analysis of all studied samples that the combination of the two most stable reference genes is the best option and the combination of the five most stable reference genes is the second-best option for accurate normalization

The geNorm software evaluates reference genes by their *M*-stability values and *V*-pairwise variability values. Low *M* values represent more stable expression and thus the most suitable reference genes (Figure 1). The geNorm analysis identified *RDN18*, *RDN25*, and *RDN5.8* as the three most stable genes; *UBC13*, *PGK1*, and *UBC7* as relatively stable genes; and *RPL13A*, *ACT1*, and *SDHA* as the three least stable genes under fluconazole treatment in *C. glabrata*. Interestingly, the ranking of expression stability of the 16 reference genes was identical between the geNorm program and the hkgFinder tool (Figure 1; Tables 3 and 4). The geNorm program also estimates the optimal number of reference genes that could be used in combination as an NF value (Figure 2). Each NF was calculated as the geometric mean of the two most stable genes, then the pairwise variability *V* was computed between NF*n* and NF*n* + 1 for *n* = 2, …, 15. Vandesompele et al. [9] proposed 0.15 as a cutoff value for *V* below which additional reference genes do not need to be added to the NF. Adding the third gene to the most stable two reference genes, *RDN18* and *RDN25*, produced a *V* below the cutoff of 0.15, indicating that it would not be necessary to include additional reference genes for normalization; the second best choice of reference gene combination based on geNorm *V* was the top five most stable genes, i.e., *RDN5.8*, *RDN18*, *RDN25*, *UBC13*, and *PGK1*. This reference gene combination showed smaller variation than other gene combinations, with a smaller *V* of 0.091 (Figure 2).

**Table 4:** Comparison of reference gene expression stability* as determined by four different software packages

**geNorm**		**BestKeeper**		**NormFinder**		**hkgFinder**	
**Gene**	**Mean** ***M***	**Gene**	***r***	**Gene**	**Stability value**	**Gene**	**Fold change**
*RDN18*	0.042	*UBC7*	0.983	*UBC7*	0.425	*RDN18*	1.2
*RDN25*	0.050	*UBC13*	0.978	*PGK1*	0.459	*RDN25*	1.3
*RDN5.8*	0.055	*PGK1*	0.974	*UBC13*	0.460	*RDN5.8*	1.4
*UBC13*	0.357	*RDN5.8*	0.915	*PPIA*	0.534	*UBC13*	2.5
*PGK1*	0.478	*RDN25*	0.908	*RDN5.8*	0.922	*PGK1*	2.9
*UBC7*	0.500	*RDN18*	0.901	*RDN25*	0.935	*UBC7*	3.0
*PPIA*	0.849	*PPIA*	0.864	*RDN18*	0.961	*PPIA*	8.7
*RPL13A*	1.103	*RPL13A*	0.857	*RPL13A*	0.993	*RPL13A*	18.0
*ACT1*	1.167	*SDHA*	0.856	*ACT1*	1.200	*ACT1*	21.8
*SDHA*	1.258	*ACT1*	0.836	*SDHA*	1.480	*SDHA*	32.7

*Genes are listed from most to least stable for all four methods.

***M***, geNorm stability parameter. **Mean*M*,** stability value.

***r*,** all 10 candidate reference genes were correlated and were combined to calculate the BestKeeper index, which was then used to determine the correlation between each reference gene and the index.

The NormFinder program was also used to rate candidate reference gene stability according to a stability value computed from the intragroup and intergroup expression variability. The least reliable reference genes identified by this program were *RPL13A*, *ACT1*, and *SDHA*, which were identical to the worst reference genes identified by geNorm and hkgFinder analyses (Table 4). However, the ranking order of the most stable genes and the relatively stable genes by the NormFinder program was different from that generated by geNorm and hkgFinder (Figure 1; Tables 3 and 4). The geNorm and hkgFinder analyses graded *RDN18*, *RDN25*, and *RDN5.8* as the most stable reference genes, followed by *UBC13*, *PGK1*, and *UBC7* based on gene expression stability, whereas NormFinder rated *UBC7*, *PGK1*, and *UBC13* as the most stable reference genes, followed by *RDN5.8*, *RDN18*, and *RDN25* (Figure 1; Tables 3 and 4).

Finally, the BestKeeper program was used to grade candidate reference gene stability. This approach permits a comparative analysis across reference genes. Ten reference genes analyzed were correlated and were combined into an index. Subsequently, the correlation between each reference gene and the index was calculated. The best correlations between the reference genes and the BestKeeper index were obtained for *UBC7*, *UBC13*, and *PGK1* (*r* = 0.983, 0.978, and 0.974, respectively; Table 4). The rankings of the top three and the last three reference genes identified by the BestKeeper program were the same as those generated by the NormFinder analysis, although the order of stability of the other reference genes differed slightly between the two programs (Table 4).

### Validation of six candidate reference genes using the comparative C_T_ method

Following the identification of the most stable reference genes from the full gene panel of 16 genes, the comparative C_T_ method was used to validate their suitability. The comparative C_T_ method, also referred to as the ΔΔC_T_ method, is a relative quantitation of gene expression between a specific target gene and a reference gene. For the comparative C_T_ method to be valid, the efficiency of the target amplification and the efficiency of the reference (internal control) amplification must be approximately equal, and this must be determined in a validation experiment.

To this end, we first determined the amplification efficiency of 10 target genes (*CDR1*, *PDH1*, *PDR1*, *SNQ2*, *YOR1*, *ERG2*, *ERG3*, *ERG4*, *ERG10*, and *ERG11*) and six reference genes (*ACT1*, *PGK1*, *RDN5.8*, *RDN18*, *UBC7*, and *UBC13*). Standard curves were generated by plotting the dilutions of the cDNA of each gene against the C_T_ values. The linear correlation coefficient (*r*^2^) for all 10 target genes and the six reference genes ranged from 0.98 to 1.0. Based on these slopes of the standard curves, the amplification efficiencies of the cDNA standards, derived from the formula E = (10^−1/slope^ −1) × 100, ranged from 94 to 119%. The C_T_ values of all 16 genes in the samples were within the range of the standard curves. Next, the ΔC_T_ (ΔC_T_ = C_T__target_ − C_T__reference_) was calculated using the C_T_ values generated from standard curve mass points (target vs. reference gene). These ΔC_T_ values were then plotted versus log_10_ input amount of cDNA to create a semi-log regression line. The slope of the resulting semi-log regression line was used as a general criterion for passing a validation experiment. In a validation experiment that passes, the absolute value of the slope of ΔC_T_ versus log_10_ input cDNA would be <;0.1, meaning the two C_T_ versus log_10_ concentration curves are nearly parallel. As seen in Table 5, our validation experiments passed for all reference genes analyzed except *RDN18*, which had an absolute value >0.1 for the slopes of ΔC_T_ versus log_10_ input cDNA for all 10 target genes evaluated. Thus, the amplification efficiency of *RDN18* was clearly different from that of the ten target genes, whereas the other five reference genes (*ACT1*, *PGK1*, *RDN5.8*, *UBC7*, and *UBC13*) had PCR efficiencies that were similar or relatively equivalent to the target amplification efficiencies (Table 5).

**Table 5:** **Validation of reference gene suitability as an internal control based on the correlation of amplification efficiency between reference and target genes in fluconazole-treated** ***C. glabrata*** **cells**

**Gene**	***CDR1***	***PDH1***	***PDR1***	***SNQ2***	***YOR1***	***ERG2***	***ERG3***	***ERG4***	***ERG10***	***ERG11***
*ACT1*	0.056	0.072	0.061	0.019	0.033	0.046	0.034	0.095	0.054	0.043
*PGK1*	0.018	0.049	0.014	0.059	0.094	0.057	0.032	0.024	0.046	0.028
*RDN5.8*	0.084	0.065	0.014	0.021	0.014	0.086	0.060	0.060	0.029	0.061
*RDN18*	0.187	0.315	0.304	0.262	0.210	0.836	0.689	0.689	0.778	0.688
*UBC7*	0.015	0.051	0.012	0.062	0.096	0.077	0.052	0.044	0.066	0.048
*UBC13*	0.069	0.051	0.028	0.035	0	0.079	0.068	0.068	0.022	0.069

Each value is the slope m of the line (Y = mx + b) of the validation experiment and reflects the correlation of reference gene and target gene amplification efficiencies.

An absolute slope value <;0.1 is generally used as a criterion for passing a validation experiment, as it indicates that the amplification efficiency is approximately equal between the reference and target genes.

The slope m = 0 indicates that the efficiencies of the two PCR reactions are equal.

### Comparison of expression levels of inducible target genes using different reference genes for normalization

To test the effect of azole on the expression of pleiotropic drug resistance genes in *C. glabrata*, we assessed the fluconazole-induced expression of two ABC genes (*CDR1* and *PDR1*) and one ERG gene (*ERG4*) in five *C. glabrata* strains, including the *PDR1* mutant strain CgB4 (Table 1). For comparison, we used both *RDN5.8* and *ACT1* as references for normalization. As shown in Table 6, fluconazole markedly induced increases in ERG4 mRNA levels in all *C. glabrata* strains examined when normalized to *RDN5.8*. Fluconazole also significantly increased CDR1 and PDR1 mRNA expression in all of the strains except CgB4, consistent with the critical role of *PDR1* in azole-induced transactivation of ABC transporter gene expression, but not ergosterol biosynthesis gene expression, in *C. glabrata*. In contrast, when using *ACT1* as the reference gene for quantification, fluconazole appeared to down-regulate the expression of all three target genes in the five *C. glabrata* strains (Table 6).

**Table 6:** **Comparison of fluconazole-induced target gene expression**^**¶**^**among five different** ***C. glabrata*** **strains using** ***RDN5.8*** **or** ***ACT1*** **as the reference for normalization**

	***CDR1***		***PDR1***		***ERG4***	
**Strain**	***RDN5.8***	***ACT1***	***RDN5.8***	***ACT1***	***RDN5.8***	***ACT1***
**NCCLS84**	2.12*	0.19*	2.22*	0.20*	2.85*	0.26*
**Cg84u**	3.90*	0.42*	3.26*	0.50*	10.04*	0.68*
**CgB4**	0.64^#^	0.06*	1.08^#^	0.10*	2.42*	0.22*
**Cg3S**	2.59*	0.23*	3.31*	0.30*	3.84*	0.35*
**Cg4R**	2.14*	0.19*	3.68*	0.33*	5.34*	0.48*

^¶^Values indicate the fold change in RNA transcription for each target gene in fluconazole-treated *C. glabrata*, as compared with untreated cells.

ΔC_T_ = C_T__target_ − C_T__reference_; ΔC_T_ of each target gene was calculated by using *RDN5.8* or *ACT1* as the reference.

ΔΔC_T_(t) = ΔC_T_(t) − ΔC_T_(ut); ut, untreated; t, fluconazole-treated. Fold change = 2^-ΔΔCT^(t).

Fold changes of the target genes were also computed with the hkgFinder tool by using *RDN5.8* or *ACT1* as the reference.

^#^*P* > 0.05 and *P <; 0.05 for the azole-treated group vs. the untreated group after normalization to *RDN5.8* or *ACT1.*

Finally, we compared the fluconazole-inducibled mRNA expression levels of four target genes (*CDR1*, *PDR1*, *ERG4*, and *ERG10*) in *C. glabrata* (Cg84u strain) after normalizing to different reference genes (*ACT1*, *PGK1*, *RDN5.8*, and *UBC13*), individually and in pairs. Differences in quantitation were detected according to the reference genes used. As seen in Table 7, normalization of the RT-qPCR data against the reference genes suggested as optimal by the four software packages (hkgFinder, geNorm, BestKeeper, and NormFinder) or the 2^-ΔΔCT^ method, gave comparable relative expression levels of the target genes under fluconazole treatment in *C. glabrata*. However, normalization against *ACT1* resulted in relative expression levels of the targets that were substantially different from those normalized using other reference genes, implying that *ACT1* is not a suitable reference gene for these studies. Taken together with the data shown above, these results demonstrate that the relative quantification of azole-inducible gene expression varies largely depending on the reference gene and the number of reference genes used for normalization. This highlights the importance of choosing a suitable reference gene or reference gene pair when using RT-qPCR to determine the level of target gene expression in this model system.

**Table 7:** Comparison of the relative mRNA expression levels* of four target genes in fluconazole-treated ***C. glabrata*** **when normalized to different reference genes**

**Gene**	***CDR1***	***PDR1***	***ERG4***	***ERG10***
***ACT1***	0.26	0.27	0.70	1.21
***RDN5.8***	4.07	4.27	11.08	19.07
***PGK1***	1.90	1.99	5.17	8.90
***UBC13***	2.23	2.33	6.06	10.43
***RDN5.8*** **+** ***PGK1***	2.79	2.92	7.59	13.06
***RDN5.8*** **+** ***UBC13***	3.01	3.15	8.19	14.09
***RDN5.8*** **+** ***PGK1*** **+** ***UBC13***	2.58	2.71	7.03	12.10

*Values indicate the fold change in RNA transcription for each target gene in fluconazole-treated *C. glabrata* after normalization to one or more reference genes, as compared with untreated cells.

ΔC_T_ = C_T__target_ − C_T__reference._

ΔΔC_T_(t) = ΔC_T_(t) − ΔC_T_(ut); ut, untreated; t, fluconazole-treated. Fold change = 2^-ΔΔCT^(t).

Fold changes of the target genes were also computed with the hkgFinder tool by using one or more reference genes as the internal control for normalization

*P* <; 0.05 vs. untreated cells, for all values shown in the table after normalizing to one or more reference genes as indicated.

## Discussion

In any gene expression study, the selection of a valid normalization or internal control gene to correct for differences in RNA sampling is critical in order to avoid misinterpretation of results and to obtain reliable conclusions. When choosing a reference gene as the internal endogenous control for gene expression studies by RT-qPCR, two important criteria must be met. The expression of the reference gene must remain stable throughout the given intervention (i.e., stability), and the amplification efficiency of the reference gene should be similar to that of the genes of interest (i.e., suitability). In the present study, we used five different methods to evaluate 16 reference genes for potential use as internal controls and found that the reference genes performed differently in terms of stability and suitability in *C. glabrata* cells upon exposure to fluconazole. To our knowledge, this is the first report to validate reference genes as RNA internal references in *C. glabrata*.

The poor performance of *ACT1* in *C. glabrata* cells was surprising, given that this gene has been used frequently as the reference gene in earlier gene expression studies [1,34,37,38]. Our data clearly demonstrate the unsuitability of *ACT1* as an internal control for gene expression studies in *C. glabrata* following fluconazole treatment. The initial results gained from using *ACT1* as the internal control suggested that target gene expression was not up-regulated (Tables 6 and 7). In fact, the only substantial change caused by azole treatment was a greater increase in *ACT1* RNA transcription compared with target gene transcription. While these findings are relevant to our specific study, it appears that numerous other studies have also shown the potential of *ACT1* to detrimentally affect the accuracy of results [11,12,15,19,26,27,29,30,32,33,45-47]. We have been using *ACT1* as the internal control for quantitation of gene expression by RT-qPCR in clinical isolates of *C. glabrata*, and we find that this gene works well as the reference in cells without azole or other agent stimulation [1]. However, Edlind and colleagues used *ACT1* as the reference gene for azole-inducible gene expression studies by slot blotting in *Candida* species, and their data clearly show the variation of *ACT1* expression in response to azoles in their systems [34,38]. Thus, our data revealing the instability of *ACT1* in *C. glabrata* following antifungal treatment, combined with evidence from mammalian and other fungus studies, add to the growing body of evidence that *ACT1* expression is unstable across various cell types and under different experimental conditions [11,12,15,26,27,29,30,32,33,45-47].

Although *ACT1* gene expression was variable in response to fluconazole, the three ribosomal RNAs (i.e., *RDN5.8*, *RDN18*, and *RDN25*) remained unaffected and showed stable expression in azole-treated *C. glabrata*. These results indicate that ribosomal RNA expression offers superior consistency compared with the expression of *ACT1* and the other reference genes assessed. The stable expression levels of 18S and 28S rRNAs relative to other reference genes under a variety of experimental conditions has previously been described for numerous systems, including both mammalian and yeast cells [10,11,15,18,23,30,48-50]. The levels of ribosomal RNA, which represents 80% of total RNA, are thought to be less likely to vary under conditions that affect the expression of mRNAs because they are transcribed by a distinct RNA polymerase. As an example, Thellin et al. and other groups have recommended the use of 18S or 28S rRNA as an internal control for mRNA quantification studies because mRNA variations are weak and cannot highly modify the total RNA level [10,11,15,18,30,39,48-52]. However, our further validation experiments showed that of slopes of ΔC_T_ versus log_10_ cDNA were sufficiently parallel between *RDN5.8* and the target genes, but not between *RDN18* or *RDN25* and the target genes. With *RDN18* and *RDN25*, the absolute slope values of the ΔC_T_ versus log_10_ input cDNA lines were >0.1 for all target genes. These data indicate that the amplification efficiency of *RDN5.8* was similar to the efficiencies of the target genes, whereas the amplification efficiencies of *RDN18* and *RDN25* were different from the target gene amplification efficiencies. This may be attributable to the much higher abundance of *RDN18* and *RDN25* than *RDN5.8* compared with target mRNA transcripts, making it difficult to accurately subtract the baseline value in RT-qPCR data analysis. Therefore, although all three ribosomal RNA subunits were stable during fluconazole stimulation, only *RDN5.8* may offer a more accurate and suitable alternative to *ACT1* as an internal control for gene expression studies in *C. glabrata*.

GAPDH, a glycolytic enzyme, is encoded by a single gene and has the advantage of being highly conserved across different species [53,54]. Like 18S rRNA and β-actin, *GAPDH* has been commonly used as an internal control, often without testing. In the present study, *GAPDH* showed much higher variability than any of the ribosomal RNAs in fluconazole-treated samples. These data demonstrate that *GAPDH* is not an appropriate control gene for these studies, as has been pointed out in previous examples, and that it may lead to incorrect results under specific experimental conditions [12-15,19,29-31,33,55]. Previous studies have indicated the instability of *GAPDH* in mammalian systems, and this study broadens the scope of this phenomenon to *C. glabrata* as well.

*PGK1* also plays important roles in the glycolytic pathway, and *PGK1* and *GAPDH* are potentially co-regulated [56]. In our data, however, their potential co-regulation was not significant. PGK1 mRNA levels remained relatively stable, in contrast to the marked variation in GAPDH mRNA levels in *C. glabrata* cells, following fluconazole challenge. Moreover, our comparative C_T_ calculations showed that the efficiency of *PGK1* amplification was approximately equal to the efficiencies of the target gene amplifications. Although *PGK1* shows some variation as a reference gene, this may not affect experimental results as long as the intergroup difference being measured is greater than the reference gene variation, that is, a reference gene RNA that has an error of 1 log_2_ may not be ideal, but it would be sufficient to measure a 2 log_2_ change in a gene of interest. Thus, it is inferred that *PGK1* may be a suitable reference gene for the analysis of expression for genes with higher azole-inducible mRNA levels, such as *ERG4* and *ERG10*.

Ubiquitin is a small regulatory protein that has been found in almost all tissues of eukaryotic organisms. The *UBC* gene codes for a polyubiquitin precursor protein [57]. Due to its ubiquitous existence in different tissues and cells in eukaryotes, there are an increasing number of studies in the literature using the *UBC* gene as the internal standard for gene expression analysis in different eukaryotic cell systems [26,29]. Out of curiosity, we validated three *UBC* genes (*UBC4*, *UBC7*, and *UBC13*) in this study. Interestingly, we found that UBC7 and UBC13 mRNAs (particularly the latter) were relatively stable in *C. glabrata* during fluconazole treatment. In addition, our validation experiments demonstrated that the amplification efficiencies of these genes were approximately equal to those of the target genes. These findings indicate that like *PGK1*, *UBC13* and *UBC7* may also be suitable internal controls for quantifying the expression of specific genes with higher azole-inducible mRNA levels in *C. glabrata*, such as some ergosterol biosynthesis genes.

To successfully select reference genes for our studies involving azole treatment, we also investigated seven other reference genes, in addition to the reference genes mentioned above, with a diversity of functions. These reference genes can be generally classified into several groups: transcription-related genes (*EF1α*), structure/cytoskeleton-related genes (*TUB1*), protein synthesis-related genes (*RPL2A*, *RPL10*, and *RPL13A*), and finally, genes that cannot be clearly categorized, including *PPIA* and *SDHA*. These potential reference genes such as *PPIA*, *RPL13A*, and *TUB1* are other examples of commonly used internal controls [28,29,32]. For example, *PPIA* has been used as a reference gene because of its remarkable evolutionary conservation and broad cellular and tissue distribution [58]. Although these seven reference genes have been used as internal standards for normalization in countless studies, all of these genes showed an unacceptable variable expression in our model system, with values ranging from a 4.4-fold induction with *TUB1* to a 23-fold induction with *SDHA* after antifungal treatment. Altogether, these results suggest that the choice of internal controls is highly specific to a particular experimental condition, thus highlighting the importance of validating reference genes for each experimental model before commencement of RT-qPCR studies.

Although it is now widely accepted that normalizing to a single reference gene represents a strategy that is simple to use and can control for every stage of the RT-qPCR, some researchers also advocate the use of two or more reference genes, rather than relying on a single RNA transcript [9,10,44,59]. This is a robust method for providing accurate normalization and is consequently preferable when fine measurements are to be made. According to Vandesompele et al. [9], the purpose of normalization is to remove the sampling difference (such as RNA quantity and quality) in order to identify real gene-specific variation. They provided evidence that a conventional normalization strategy based on a single gene can lead to erroneous normalization. However, it is not always possible to measure multiple reference genes because of limited sample availability and cost. Furthermore, even when multiple genes are chosen, the resolution of the particular assay remains dependent on the variability of the chosen reference genes. As to our case, the geNorm analysis using the geometric mean of the expression of the 16 candidate cDNAs suggested the use of *RDN5.8*, *RDN18*, and *RDN25* in combination or the combination of these three ribosomal RNAs plus *UBC13* and *PGK1* as the reference control in the current study. However, the geNorm assessment is based solely on the variability of reference genes and does not take other factors into account. For example, we found that although *RDN18* and *RDN25* were quite stable, their amplification efficiencies were not equal to the amplification efficiencies of all target genes tested; thus, they may not be suitable as internal controls in our system. Therefore, when multiple reference genes are necessary, we believe that the combination of *RDN5.8* plus *UBC13* and/or *PGK1* would be a better choice for quantitation of gene expression by RT-qPCR in *C. glabrata* following azole stimulation.

## Conclusions

In this study, we evaluated 16 reference genes for potential use as internal controls for RT-qPCR analysis of gene expression in *C. glabrata* (Cg84u strain) following 2 hours of exposure to fluconazole at 200 μg/ml. To our knowledge, this is the first identification and validation of *RDN5.8*, *UBC13*, and *PGK1* as the most suitable and stably expressed reference genes among the 16 reference genes tested. Therefore, we recommend the use of *RDN5.8*, *UBC13*, or *PGK1* alone or the geometric mean of these genes as standards for normalization when analyzing differences in gene expression levels in *C. glabrata* during antifungal treatment. More specifically, *RDN5.8* may be a more suitable reference gene for the analysis of expression for genes with lower azole-inducible mRNA levels, while *UBC13* and *PGK1* may be better internal controls for quantifying the expression of genes with higher azole-inducible mRNA levels in *C. glabrata*. In contrast, we demonstrated that 10 reference genes commonly used in published reports, including *ACT1*, *GAPDH*, *PPIA*, *RPL13A*, and *TUB1*, had significant differences in their expression upon azole challenge, and thus were not validated as good endogenous controls in this model. As a main conclusion, this study emphasizes the importance of evaluation studies for the selection of the most appropriate internal controls for each experimental model used for quantitative expression studies.

## Abbreviations

ACT1: beta-actin (β-actin); bp: Base pair; cDNA: Complementary DNA; Cg: Candida glabrata; CP: Crossing point; ΔC_T_: Delta cycle threshold; CV: Coefficient of variation; EF1α: Elongation factor 1α; FC, fold change; GAPDH: Glyceraldehyde 3-phosphate dehydrogenase; M: GeNorm stability parameter; MIC: Minimum inhibitory concentration; mRNA: Messenger RNA; NF: Normalization factor; ORF: Open reading frame; PCR: Polymerase chain reaction; PGK1: Phosphoglycerate kinase; PPIA: Peptidylpropyl isomerase A (cyclophilin A); RDN5.8: 5.8 S ribosomal RNA; RDN18: 18S ribosomal RNA; RDN25: 25S ribosomal RNA; RPL2A: Ribosomal protein, large, 2A; RPL10: Ribosomal protein, large, 10; RPL13A: Ribosomal protein, large, 13A; RT: Reverse transcription; RT-qPCR: Real-time quantitative reverse-transcription PCR; SD: Standard deviation; SDHA: Succinate dehydrogenase complex, subunit A; Tm: Melting temperature; TUB1: Alpha-tubulin (α-tubulin); UBC: Ubiquitin C; V: geNorm pairwise variation.

## Competing interests

The authors declare that they have no competing interests.

## Availability and requirements

The hkgFinder software is currently available as an R source script and it can be downloaded for free from the NIAID Exon website [ http://exon.niaid.nih.gov/hkgFinder/] with a sample data set and complete instructions. It requires installation of R 2.11 or higher on computers using the Microsoft Windows operating system for complete compatibility with its graphic user interface (GUI) elements. Experienced R users should be able to use hkgFinder on an Apple Macintosh or Linux/Unix operating system with some reasonable adjustments. An hkgFinder webtool should be available soon on the NIAID Exon website listed above. The geNorm software is available from Biogazelle [ http://www.biogazelle.com/genormplus/] with a free 15-day trial download as a part of the qBase PLUS software system. The geNorm software has now been integrated into the qBase PLUS software, where calculation of relative quantities and geNorm analysis are combined in a single program to speed up analysis. Presently, many manual pre-calculations are not needed, and cross point (CP) values from RT-qPCR can now be directly used for the gene stability analysis using the qBase PLUS software. The qBase PLUS software currently requires Microsoft Windows XP or above or Apple Mac OS X 10.6 (Snow Leopard) with Java 1.6 or later. Support for Linux is also available, but no requirements are listed on the manufacturer’s website. The BestKeeper software [ http://gene-quantification.com/bestkeeper.html] is available for free download. Please note that the software requires a password generated by an automatic email response from genequan@wzw.tum.de or password@gene-quantification.info. It requires the Microsoft Windows operating system and Microsoft Excel, but specific versions are not listed on the manufacturer’s website. The NormFinder software is available for free download from the manufacturer’s website [ http://www.mdl.dk/publicationsnormfinder.htm]. It requires Microsoft Windows operating system and Microsoft Excel 2003 or above.

## Authors’ contributions

QQL conceived of the project, conducted the studies, performed all the experimental procedures, carried out the analysis and interpretation of data, wrote the manuscript, and is the primary author of this paper. JS developed the hkgFinder software and technically helped with the use of other software packages in the present study. JEB participated in the design and coordination of the study and critically reviewed the manuscript. All authors have read and approved the final manuscript.

## Supplementary Material

Additional file 1 Summary of the reference genes evaluated in this study. Click here for file

Additional file 2 Primers and probes for RT-qPCR analyses of reference gene RNA transcription in this study. Click here for file

Additional file 3 Primers and TaqMan probes for RT-qPCR analyses of target gene expression in this study. Click here for file

Additional file 4 hkgFinder. Click here for file
