# Supplementary material for: Evaluation of reference genes for real-time quantitative PCR studies in Candida glabrata following azole treatment
Source: BMC Mol Biol. 2012 Jun 29;13:22. doi: 10.1186/1471-2199-13-22 (PMC3482582; doi:10.1186/1471-2199-13-22)
Supplement: Additional file 1 — Summary of the reference genes evaluated in this study. [file 1471-2199-13-22-S1.doc]

**Additional table 1. Summary of the reference genes evaluated in this study**

**______________________________________________________________________________**

#### Symbol Gene number* Gene name Gene function

**______________________________________________________________________________**

*RDN5.8* CAGL0L13387r 5.8S ribosomal RNA Structural constituent of ribosome, involved in translation

*RDN18* CAGL0L13398r 18S ribosomal RNA Structural constituent of ribosome, involved in translation

*RDN25* CAGL0L13365r 25S ribosomal RNA Structural constituent of ribosome, involved in translation

*ACT1* CAGL0K12694g Beta-actin (β-actin) Structural constituent of cytoskeleton, involved in cell motility, polarization, endocytosis, and other functions

*EF1α* CAGL0K07832g Elongation factor 1α Plays a central role in translation process and nuclear export of proteins

CAGL0D01188g

*GAPDH* CAGL0G09383g Glyceraldehyde-3-phosphate Oxidoreductase in glycolysis and gluconeogenesis,

CAGL0J00451g dehydrogenase phosphorylation of glyceraldehyde-3-phosphate

*PGK1* CAGL0L07722g Phosphoglycerate kinase Glycolytic enzyme

*PPIA* CAGL0E01177g Peptidylpropyl isomerase A Serine-threonine phosphatase inhibitor

(cyclophilin A)

*RPL2A* CAGL0J02354g Ribosomal protein, large, 2A Structural component of the large 60S ribosomal subunit, catalysis of protein synthesis

*RPL10* CAGL0K12826g Ribosomal protein, large, 10 Structural component of the large 60S ribosomal subunit, catalysis of protein synthesis

*RPL13A* CAGL0L06886g Ribosomal protein, large, 13A Structural component of the large 60S ribosomal subunit, catalysis of protein synthesis

*SDHA* CAGL0I04598g Succinate dehydrogenase complex, Electron transporter in TCA cycle and respiratory chain,

subunit A, flavoprotein involved in oxidation of succinate

*TUB1* CAGL0B02497g Alpha-tubulin (α-tubulin) Cytoskeletal structural protein

*UBC4* CAGL0E04752g Ubiquitin C4 Ubiquitin-protein ligase activity, involved in ER-associated protein catabolism

*UBC7* CAGL0M07568g Ubiquitin C7 Ubiquitin-protein ligase activity, involved in ER-associated protein catabolism

*UBC13* CAGL0G08063g Ubiquitin C13 Ubiquitin-protein ligase activity, involved in ER-associated protein catabolism

______________________________________________________________________________

***Genolevures: Yeast Genomes [www.genolevures.org/yeastgenomes.html]**
