# Supplementary material for: Evaluation of reference genes for real-time quantitative PCR studies in Candida glabrata following azole treatment
Source: BMC Mol Biol. 2012 Jun 29;13:22. doi: 10.1186/1471-2199-13-22 (PMC3482582; doi:10.1186/1471-2199-13-22)
Supplement: Additional file 2 — Primers and probes for RT-qPCR analyses of reference gene RNA transcription in this study. [file 1471-2199-13-22-S2.doc]

Additional table 2. Primers and probes for RT-qPCR analyses of reference gene RNA transcription in this study

______________________________________________________________________________

#### Gene Primer and probe sequence (5′→3′) Amplicon size (bp) Tm (°C)

#### ______________________________________________________________________________

*RDN5.8* F: CTTGGTTCTCGCATCGATGA 98 77

R: GGCGCAATGTGCGTTCA

P: ACGCAGCGAAATGCGATACGTAATGTG

*RDN18* F: AGAAACGGCTACCACATCCAA 100 80

R: CGAATGGGCCCTGTATCGT

P: CAGCAGGCGCGCAAATTACCCA

*RDN25* F: AACAACTCACCGGCCGAAT 102 81

R: CCTACTCGTCAGGGCCTCATT

P: CAAGCGTGTTACCTATACTCCGCCGTCA

*ACT1* F: TTACCAACTGGGATGACATGGA 100 79

R: GGAGCCTCGGTCAACAAGAC

P: CACTTTCTACAACGAATTGAGAGTCGCCCC

*EF1α* F: GAGTCGAAACCGGTGTCATCA 100 80

R: AATTGTTCGTGGTGCATTTCAA

P: TGGTTGTTACCTTCGCCCCAGCTG

*GAPDH* F: AACCGCTTCCGGTAACATCA 101 81

R: TGAAAGCCATACCGGTCAACT

P: CATCCTCCACCGGTGCCGCT

*PGK1* F: CAAACGGTGAAAGAAACGAGAA 100 78

R: CCGACACAGTCGTTCAAGAAAG

P: TCCTTGGCTCCAGTTGCTGAAGAGCTAC

*PPIA* F: GACGAGAACTTTGTCAAGAGACATG 100 80

R: GCACGGTGGTGATGAAGAATT

P: TGGCCAACGCCGGTCCAAA

*RPL2A* F: GCCGGTAAGAAGGCCTCTTT 101 79

R: CTGTCACCTGGCTTTTCTTCAA

P: CGTCTTGCCATTGGGTTCCGTTCC

*RPL10* F: GAGATTCTTTCCACTTGAGAGTCAGA 105 79

R: CTCTCATACCTTGTTGCAATCTATCC

P: CACCCATTCCACGTCTTGAGAATCAACA

*RPL13A* F: ACCAAGCTGGTAAGAAGGAATCC 103 79

R: GGAGCTCTAACGACTGGTCTCAA

P: CTGCCAAGATCGCTCCAAGACCATTG

*SDHA* F: CATTGCATGTTGGTGGCTACA 125 76

R: GGCAGAGCCATTATTGGGAAT

P: TTCAACAATTTTCTTTGCCGCGCTATTG

*TUB1* F: ACCTTCATGGTCGACAACGAA 100 81

R: CCTGGGCGACCAAGTTATTC

P: CAAGAAGAACCTGGGCATCACCAGACC

*UBC4* F: CCAGTGGTCTCCAGCTCTAACA 100 80

R: GGCGATCTCTGGAACCAAAG

P: TCCTGCTTTCCATCTGCTCCTTGCTG

*UBC7* F: CTACACCCTAATATCTACCCGAATGGT 100 77

R: ACCATCTCTCCTCTGCAGACTCA

P: TGCATATCGATCCTGCACTCCCCAG

*UBC13* F: TGCCCGAGGACTACCCTATG 100 81

R: AGCACGTCCAGGCAGATACG

P: CCAAGATATACCACCCAAACATCGACAGGC

______________________________________________________________________________

#### F, forward primer; R, reverse primer; P, TaqMan probe, labeled as 5′-FAM, 3′-TAMRA.
