# Supplementary material for: Evaluation of reference genes for real-time quantitative PCR studies in Candida glabrata following azole treatment
Source: BMC Mol Biol. 2012 Jun 29;13:22. doi: 10.1186/1471-2199-13-22 (PMC3482582; doi:10.1186/1471-2199-13-22)
Supplement: Additional file 3 — Primers and TaqMan probes for RT-qPCR analyses of target gene expression in this study. [file 1471-2199-13-22-S3.doc]

**Additional table 3. Primers and TaqMan probes for RT-qPCR analyses of target gene expression in this study**

______________________________________________________________________________

## Gene Primer and probe sequence (5′→3′) Gene number*

______________________________________________________________________________

*ACT1* F: TTGGACTCTGGTGACGGTGTTA CAGL0K12694g

R: AAAATAGCGTGTGGCAAAGAGAA

P: CCACGTTGTTCCAATTTACGCCGG

*PDR1* F: AACGATTATTCAATTGCAACAACG CAGL0A00451g

R: CCTCACAATAAGGAAAGTCTGCG

P: TCGAATATTATGCACCATCATGTCTGTGTTTAGCT

*CDR1* F: AGATGTGTTGGTTCTGTCTCAAAGAC CAGL0M01760g

R: CCGGAATACATTGACAAACCAAG

P: TTATCTGCTGCGATGGTTCCTGCTTCC

*PDH1* F: AATGGATGTTAGAAGTAGTTGGAGCAG CAGL0F02717g

R: TGTTCGGAATTTCTCCACACCT

P: CAGGCTCACATGCAAACCAAGACTACCAT

*SNQ2* F: GCGGAAGATCGCACGAAG CAGL0I04862g

R: GGCGCGAGCGGGATA

P: CCGATGGTGACGATGCGCACAG

*YOR1* F: CGCTGGGAAGGCCAAGA CAGL0G00242g

R: CTCCCCGGACGTCAGAATAG

P: CTCGCCGGTGCAGGATTACGATCTAGA

*ERG2* F: TCCCAGGTATGACCCATCATC CAGL0L10714g

R: TGCGAAGGAGTTTTGATCCAT

P: ACAAAAGGGCTACGCAAAGCAATACGC

*ERG3* F: TGCACTGGCCTCGTGTCTAC CAGL0F01793g

R: TAACCGTCGACTGGGTGGAA

P: TGGTTGGTCTGCACTCCATTCGCC

*ERG4* F: CCCTCAATTAGGTGTCGTCATGT CAGL0A00429g

R: GGCACGATTAATTCTTCACCCTTA

P: CCACTGGCTGTACGCTAACGCTTGTG

*ERG10* F: GCCAGAACCCCAATTGGTT CAGL0L12364g

R: TGCAATGACACCTAGGTCAACAG

P: TTCCAAGGTGCGTTGGCCTCCA

*ERG11* F: TGTCTTGATGGGTGGTCAACA CAGL0E04334g

R: CTGGTCTTTCAGCCAAATGCA

P: CTTCCGCTGCTACCTCCGCTTGG

______________________________________________________________________________

**F, forward primer; R, reverse primer; P, TaqMan probe, labeled as 5′-FAM, 3′-TAMRA.**

***Genolevures: Yeast Genomes [www.genolevures.org/yeastgenomes.html]**
