# Supplementary material for: Evaluation of reference genes for real-time quantitative PCR studies in Candida glabrata following azole treatment
Source: BMC Mol Biol. 2012 Jun 29;13:22. doi: 10.1186/1471-2199-13-22 (PMC3482582; doi:10.1186/1471-2199-13-22)
Supplement: Additional file 4 — hkgFinder. [file 1471-2199-13-22-S4.zip › hkgFinder/hkgFinder -- User's Manual.pdf]

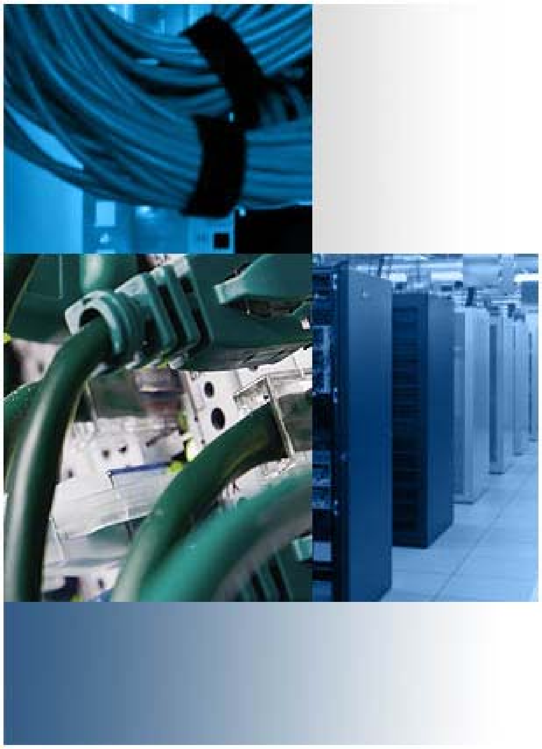

hkgFinder

Identify Housekeeping Genes and Compute Differential Expression

User's Manual

October 2011

Prepared by
Jeff Skinner, M.S.
Contractor, Lockheed Martin
Biostatistics Specialist

Table of Contents

Before You Start! 3

Using the hkgFinder script 4

1.0 Organize and Properly Format Your Data 4

2.0 Open R and Run the Script 6

3.0 Identify a Housekeeping Gene 7

4.0 Test Genes for Differential Expression 11

Before You Start!

Thank you for using this software. The hkgFinder script can be used to identify one housekeeping gene from a table of quantitative real time polymerase chain reaction (qRT PCR) data, then compute the standard Student's T-test analysis to compare delta delta CT and Fold Change differences between two types of samples. Please follow these instructions to use the software:

- Please install The R Project for Statistical Computing (http://www.r-project.org/) software on your computer. Visit http://cran.r-project.org/ for download links and instructions.
- Please save the zip file "qRTpcr.zip" in a safe location (e.g. your personal H:/ drive or desktop). You may need to access this file again if any component of the software becomes lost
- Please unzip "hkgFinder.zip" and store the resulting file folder named "hkgFinder" in a safe location. You will need access to this file folder every time you use the software. Right-click over the hkgFinder.zip file and select > **WinZip** > **Extract to here** if you have already pasted the zip file in a safe location.

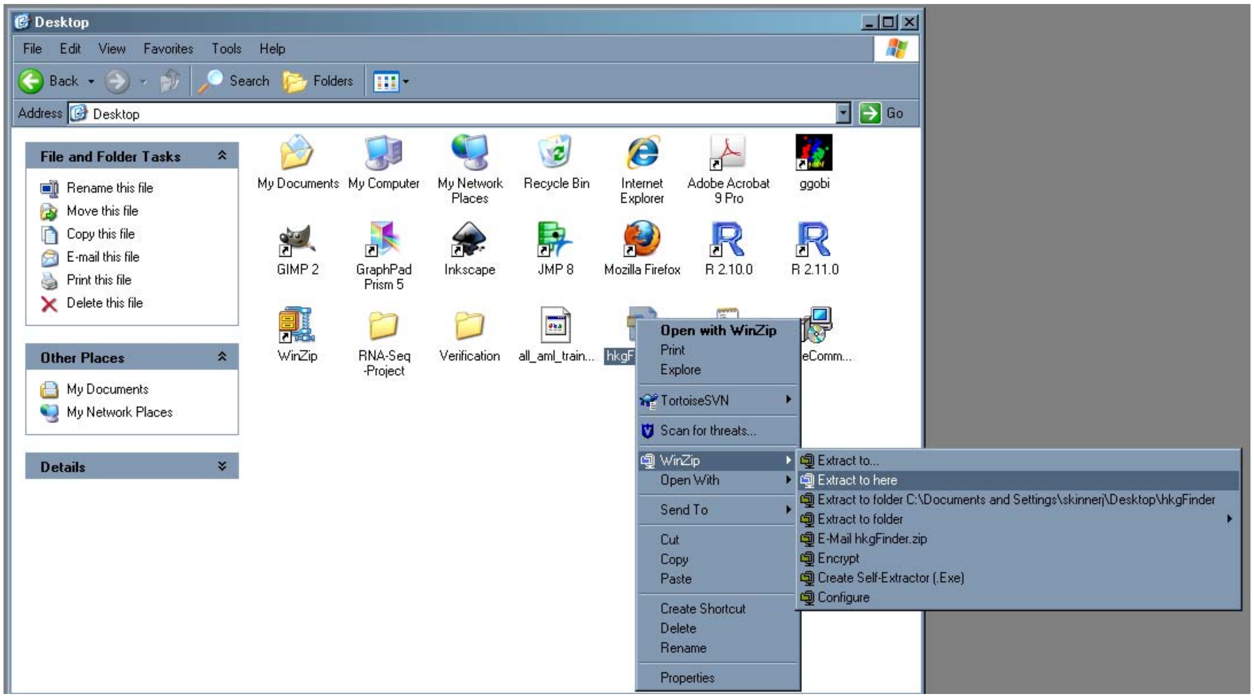

Using the hkgFinder script

1.0 Organize and Properly Format Your Data

1.1. **Properly Format Your Data**. You can use hkgFinder with Applied Biosystems 7500 Real-Time PCR System data in its native .CSV text file format. If you do not use AB7500 data, then you can convert your qRT PCR data into the correct format as shown in the SampleData.txt file.

Take your actual qRT PCR data files as shown below

05-8-2011(1).csv

	A	B	C	D	E	F	G	H	I	J	K
1	Document	Name: 05-8-2011(1).sds									
2	Plate Type: Absolute Quantification										
3	User: LCID7500										
4											
5	Document Information										
6											
7	Operator: LCID7500										
8	Run Date:	May 08	2011 16:19:47								
9	Last Modi	May 08	2011 18:27:52								
10	Instrument Type: Applied Biosystems 7500 Real-Time PCR System										
11											
12	Comments:										
13	SDS v1.3.1										
14											
15	Thermal Cycler Profile										
16	Stage	Repetitior	Temperat	Time	Ramp Rat	Auto Increment					
17	1	1	50.0 °C	2:00	100						
18	2	1	95.0 °C	10:00	100						
19	3	45	95.0 °C	0:15	100						
20			60.0 °C	1:00	100						
21	4 (Dissocia	1	95.0 °C	0:15	Auto						
22			60.0 °C	1:00	Auto						
23			95.0 °C	0:15	Auto						
24	Standard 7500 Mode										
25	Data Collection : Stage 3 Step 1										
26	PCR Volume: 25 µL										
27											
28	Well	Sample N	Detector	Task	Ct	StdDev Ct	Qty	Mean Qty	StdDev Qt	Filtered	Tm
29	A1	84u cDNA	Cg5.8S rRN	Standard	14.71	0.09					77.3
30	A2	84u cDNA	Cg5.8S rRN	Standard	14.84	0.09					77.3
31	A3	84u cDNA	Cg5.8S rRN	Standard	14.67	0.09					77.6
32	A4	84u FC cDI	Cg5.8S rRN	Unknown	14.29	0.015					77.6
33	A5	84u FC cDI	Cg5.8S rRN	Unknown	14.27	0.015					77.6
34	A6	84u FC cDI	Cg5.8S rRN	Unknown	14.27	0.015					77.6
35	A7	84u cDNA	CgRPL2A	Standard	27.05	0.02					79
36	A8	84u cDNA	CgRPL2A	Standard	27.03	0.02					79
37	A9	84u cDNA	CgRPL2A	Standard	27.07	0.02					79
38	A10	84u FC cDI	CgRPL2A	Unknown	23.22	0.167					79
39	A11	84u FC cDI	CgRPL2A	Unknown	23.37	0.167					78.7
40	A12	84u FC cDI	CgRPL2A	Unknown	23.55	0.167					78.7
41	B1	84u cDNA	Cg18S rRN	Standard	13.66	0.03					79.7
42	B2	84u cDNA	Cg18S rRN	Standard	13.61	0.03					79.7
43	[illegible]	[illegible]	[illegible]	[illegible]	[illegible]	[illegible]					[illegible]

Delete all the unnecessary header information from the file (i.e. delete rows 1 to 27). You can also delete any empty columns (e.g. Qty, Mean Qty, StdDev Qty, Filtered), but please do NOT change any column names. The resulting file should look like:

qRT PCR.txt

	A	B	C	D	E	F	G	H
1	Well	Sample Name	Detector	Task	Replicate	Ct	StdDev Ct	Tm
2	A1	84u cDNA	Cg5.8S rRN	Standard	1	14.71	0.09	77.3
3	A2	84u cDNA	Cg5.8S rRN	Standard	1	14.84	0.09	77.3
4	A3	84u cDNA	Cg5.8S rRN	Standard	1	14.67	0.09	77.6
5	A4	84u FC cDNA	Cg5.8S rRN	Unknown	1	14.29	0.015	77.6
6	A5	84u FC cDNA	Cg5.8S rRN	Unknown	1	14.27	0.015	77.6
7	A6	84u FC cDNA	Cg5.8S rRN	Unknown	1	14.27	0.015	77.6
8	A7	84u cDNA	CgRPL2A	Standard	1	27.05	0.02	79
9	A8	84u cDNA	CgRPL2A	Standard	1	27.03	0.02	79
10	A9	84u cDNA	CgRPL2A	Standard	1	27.07	0.02	79
11	A10	84u FC cDNA	CgRPL2A	Unknown	1	23.22	0.167	79
12	A11	84u FC cDNA	CgRPL2A	Unknown	1	23.37	0.167	78.7
13	A12	84u FC cDNA	CgRPL2A	Unknown	1	23.55	0.167	78.7
14	B1	84u cDNA	Cg18S rRN	Standard	1	13.66	0.03	79.7
15	B2	84u cDNA	Cg18S rRN	Standard	1	13.61	0.03	79.7

1.2. **Create a Working Directory**. To keep things simple, you will want to create a new file folder to store your data and output. Choose a safe location to store your qRT PCR data and statistical results. (e.g. H:\drive, Desktop, …).

Right-click and choose **> New > New Folder** to create a folder specifically for this data.

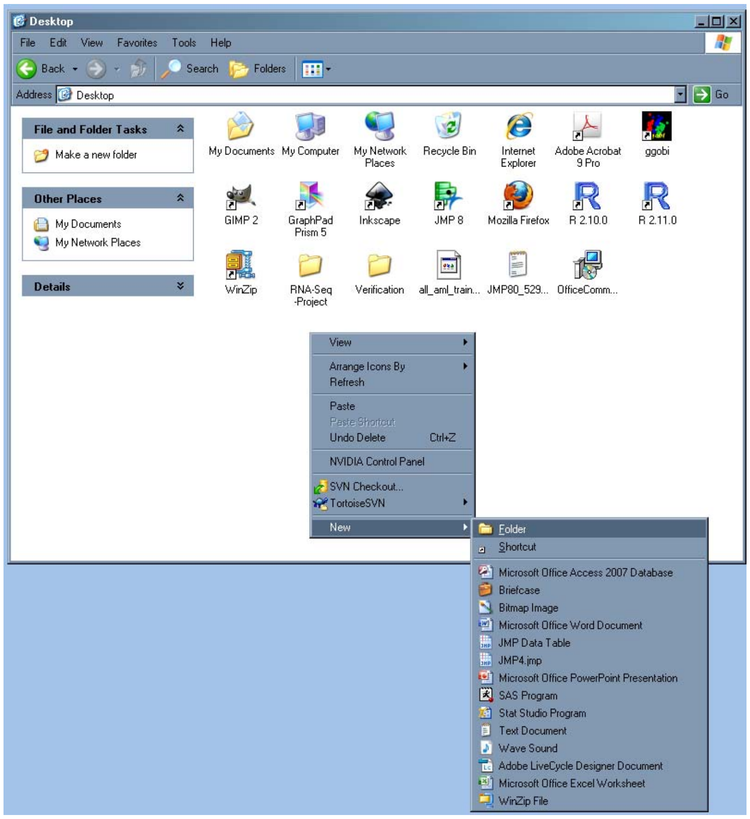

Rename the new folder and paste your re-formatted data files into the folder.

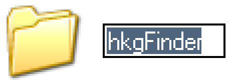

2.0 Open R and Run the Script

2.1. **Open the R software application**. Double click the R icon on your computer

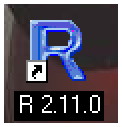

The R interface should look like this:

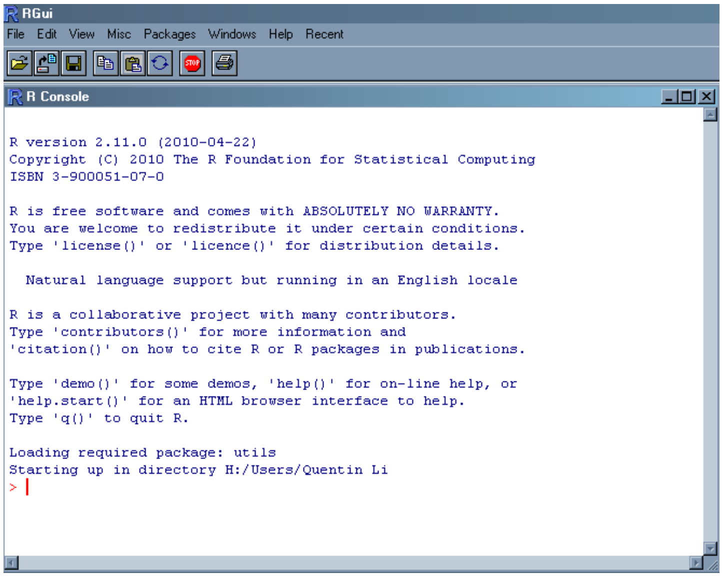

2.2. **Run the hkgFinder.R source code**. Click **> File > Source R code…** and select the hkgFinder.R file from the browse window. Click "Open" to run the program.

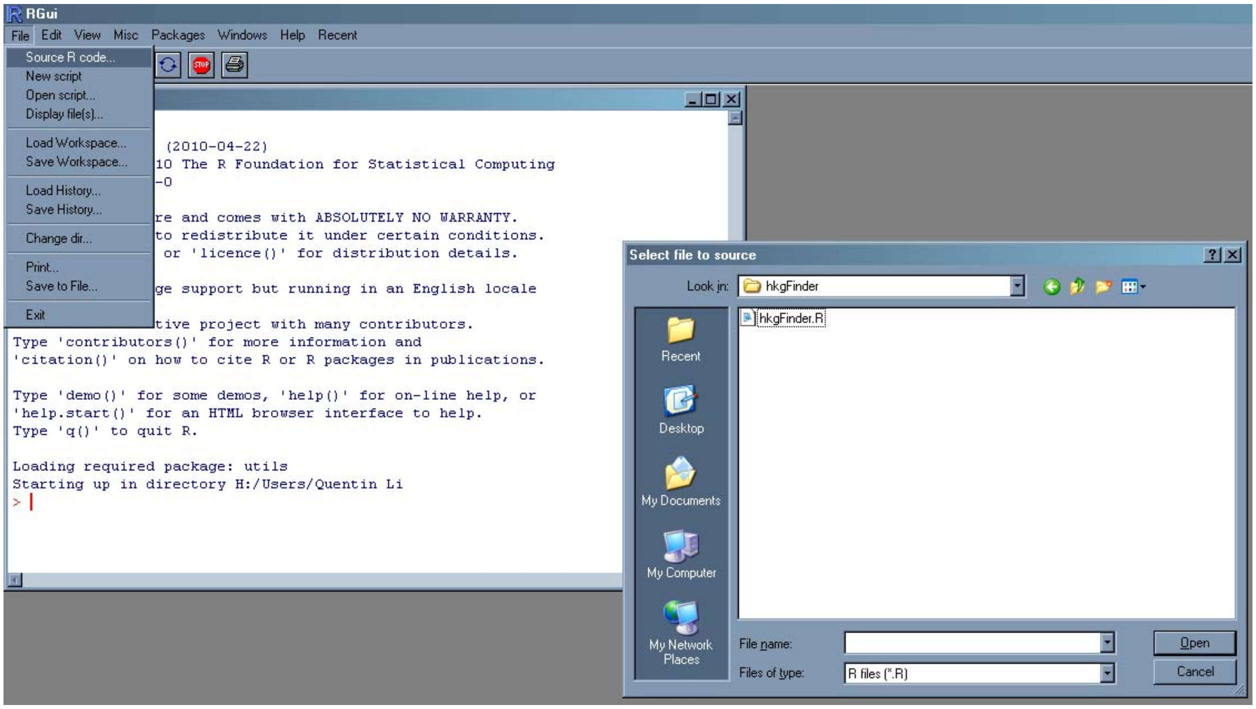

Follow the resulting pop-up Windows as shown in steps 3- below to run the program

3.0 Identify a Housekeeping Gene

3.1. **Define Your Working Directory**. After you source the hkgFinder.R code, you will immediately see the "Browse For Folder" pop-up window:

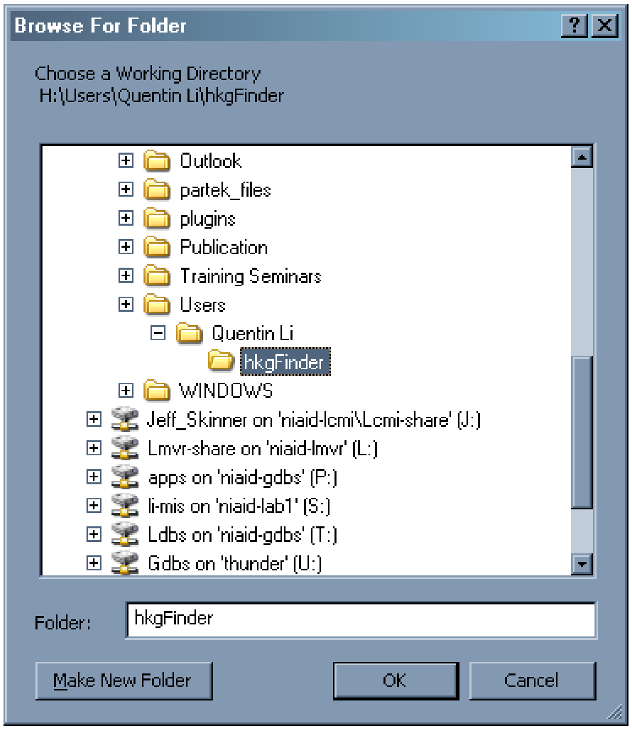

Find and select the folder containing your qRT PCR data created in step 1. E.g. > **My Computer** > **H:\drive** > **Users** > **Quentin Li** > **hkgFinder**.

3.2. **Select Your Input File Type**. Once you have chosen your working directory, you will see a pop-up menu asking you to select the input file type. You can choose to import a .CSV file from the AB7500 platform, or you can choose to import a properly formatted tab-delimited .TXT file similar to the SampleData.txt file.

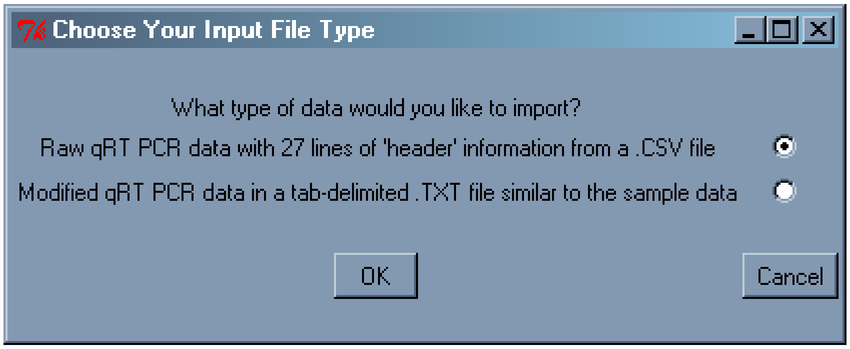

3.3. **Select Your Data File**. Immediately after you choose the working directory, you will see the "Select File" window that allows you to choose your qRT PCR data set.

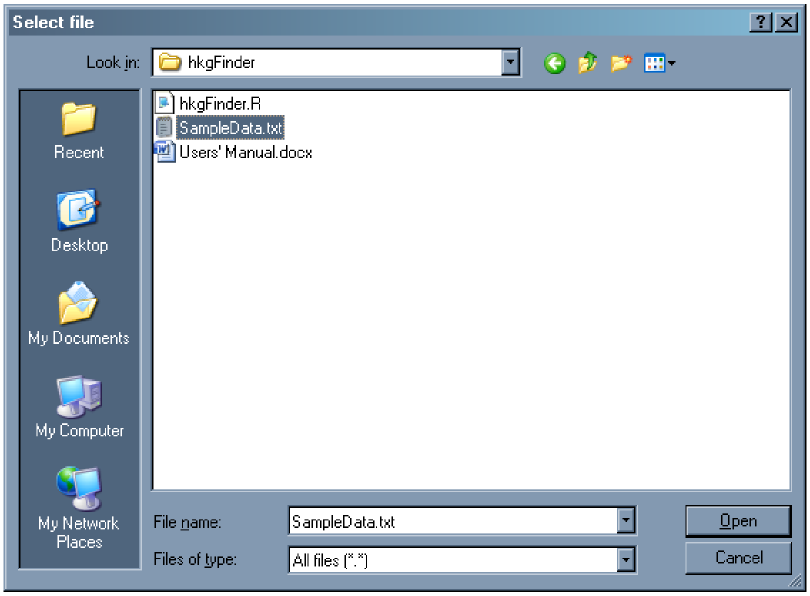

Note: You can always select the sample data set "SampleData.txt" to test the software

3.4. **Define Your TREATED Group**. After you select your qRT PCR data file, you will see a pop-up window titled "Choose a Treated Group".

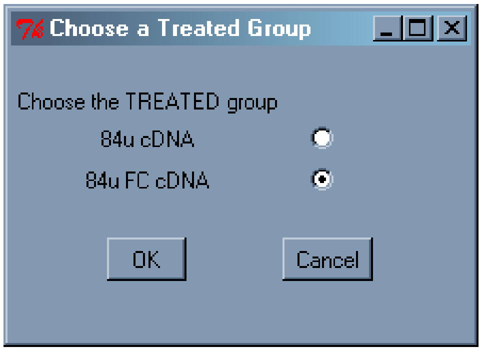

Choose the name of your TREATED group from one of the two radio buttons. Note that all fold change values will be computed as TREATED / baseline (e.g. 84u FC cDNA / 84u cDNA).

3.5. Choose a House Keeping Gene. Once the TREATED group has been chosen, your web-browser will open to display the List of Potential Housekeeping Genes.

List of Potential Housekeeping Genes

	gene	SD	logFoldChange	FoldChange	p	p.adj
3	Cg18S rRNA	0.19	-0.35	1.3	0.0001669168	0.0015
5	Cg25S rRNA	0.22	-0.39	1.3	0.0000431393	0.0006
1	Cg5.8S rRNA	0.26	-0.46	1.4	0.0110299451	0.0154
20	CgUBC13	0.73	-1.33	2.5	0.0000964116	0.0010
15	CgPGK1	0.87	-1.56	3.0	0.0077052095	0.0154
18	CgUBC7	0.88	-1.60	3.0	0.0000607197	0.0007
6	84U CgCDR1	1.14	-2.14	4.4	0.0000118594	0.0002
8	84U CgPdr1	1.19	-2.23	4.7	0.0000207900	0.0003
13	CgGAPDH	1.19	-2.18	4.5	0.0000030045	0.0001
26	CgUBC4	1.33	-2.41	5.3	0.0003017505	0.0024
24	CgTubulin-a	1.42	-2.60	6.0	0.0000680114	0.0007
16	84U CgERG11	1.47	-2.74	6.7	0.0000011710	0.0000
25	CgTubulin-b	1.52	-2.76	6.8	0.0010412835	0.0035
11	CgEF1a	1.58	-2.89	7.4	0.0000196494	0.0003
22	CgTBP	1.60	-2.92	7.6	0.0006692369	0.0035
19	CgRPP2B	1.69	-3.09	8.5	0.0000295271	0.0004
9	CgCyclophilin	1.71	-3.12	8.7	0.0000003393	0.0000
23	CgTFRC	1.90	-3.46	11.0	0.0000284112	0.0004
12	84U CgERG4	1.94	-3.68	12.8	0.0000000012	0.0000
10	84U CgERG2	1.97	-3.72	13.2	0.0000007344	0.0000
2	CgRPL2A	2.01	-3.67	12.7	0.0005750691	0.0035
4	CgRPL10	2.26	-4.12	17.4	0.0000006542	0.0000
14	84U CgERG10	2.27	-4.31	19.8	0.0000018917	0.0000
17	CgRPL13B	2.28	-4.17	18.0	0.0000004577	0.0000
7	84U CgACT1	2.44	-4.45	21.8	0.0006353703	0.0035
21	CgSdha	2.76	-5.03	32.7	0.0003919347	0.0027

The best housekeeping genes will have the smallest SD (standard deviation) and the smallest Fold Change values. The List of Potential Housekeeping Genes will already be sorted to put the best housekeeping genes at the top of the table and the worst housekeeping genes at the bottom of the table.

When the List of Potential Housekeeping Genes appears, you should also see a pop-up window asking you to choose one housekeeping gene.

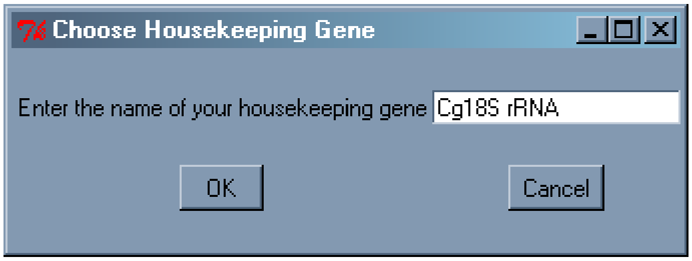

The top ranked house-keeping gene from the table will already be entered in the pop-up window (e.g. Cg18S rRNA), but you can type in the name of a different gene if you prefer.

If you enter an incorrect gene name, the software will ask you to

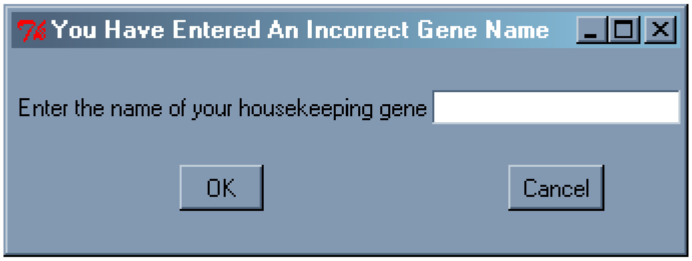

Please note that this software is case-sensitive, so “Cg18S rRNA” is different from “cg18s rrna” or “CG18S RRNA”.

If you click “Cancel” the analysis should stop.

4.0 Test Genes for Differential Expression

4.1. **Read the Final Statistical Results**. After you have chosen your housekeeping gene, another web-browser page or tab should open to display the Final Statistical Results table.

Final Statistical Results

	gene	ddCt	FoldChange	p	p.adj
11	84U CgERG4	-3.68	12.8	0.0000000012	0.0000
8	CgCyclophilin	-3.12	8.7	0.0000003393	0.0000
16	CgRPL13B	-4.17	18.0	0.0000004577	0.0000
4	CgRPL10	-4.12	17.4	0.0000006542	0.0000
9	84U CgERG2	-3.72	13.2	0.0000007344	0.0000
15	84U CgERG11	-2.74	6.7	0.0000011710	0.0000
13	84U CgERG10	-4.31	19.8	0.0000018917	0.0000
12	CgGAPDH	-2.18	4.5	0.0000030045	0.0001
5	84U CgCDR1	-2.14	4.4	0.0000118594	0.0002
10	CgEF1a	-2.89	7.4	0.0000196494	0.0003
7	84U CgPdr1	-2.23	4.7	0.0000207900	0.0003
22	CgTFRC	-3.46	11.0	0.0000284112	0.0004
18	CgRPP2B	-3.09	8.5	0.0000295271	0.0004
17	CgUBC7	-1.60	3.0	0.0000607197	0.0007
23	CgTubulin-a	-2.60	6.0	0.0000680114	0.0007
19	CgUBC13	-1.33	2.5	0.0000964116	0.0010
3	Cg18S rRNA	-0.35	1.3	0.0001669168	0.0015
25	CgUBC4	-2.41	5.3	0.0003017505	0.0024
20	CgSdha	-5.03	32.7	0.0003919347	0.0027
2	CgRPL2A	-3.67	12.7	0.0005750691	0.0035
6	84U CgACT1	-4.45	21.8	0.0006353703	0.0035
21	CgTBP	-2.92	7.6	0.0006692369	0.0035
24	CgTubulin-b	-2.76	6.8	0.0010412835	0.0035
14	CgPGK1	-1.56	3.0	0.0077052095	0.0154
1	Cg5.8S rRNA	-0.46	1.4	0.0110299451	0.0154

Read this table to identify genes that are differentially expressed between the two sample types.

See the below for details:

- Delta Ct values = Gene Ct – Housekeeping Gene Ct.
- Delta Delta Ct values = TREATED Delta Ct – baseline Delta Ct
  E.g. ddCt = 84u FC cDNA dCt - 84u cDNA dCt
- Fold Change = 2^(-Delta Delta Ct)
- P-values are computed from a simple Student's T-test
- Adjusted p-values use Holm's adjustment for multiple testing

4.2. **Save Your Final Statistical Results**. When the Final Statistical Results table appears in your web-browser, you should also see a pop-up window titled "Choose a Results File Name".

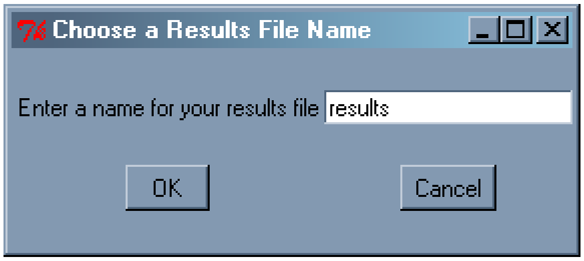

Enter a file name to save the final statistical results as a comma-separated value text file in your working directory folder.

4.3. **Analyze more data**. You can repeat steps 1.1 through 4.2 to analyze other data sets or to re-test data with a different housekeeping gene.

If you have any questions about the hkgFinder tool, please contact: ScienceApps@niaid.nih.gov.
