# Supplementary material for: Evaluation of reference genes for real-time quantitative PCR studies in Candida glabrata following azole treatment
Source: BMC Mol Biol. 2012 Jun 29;13:22. doi: 10.1186/1471-2199-13-22 (PMC3482582; doi:10.1186/1471-2199-13-22)
Supplement: Additional file 4 — hkgFinder. [file 1471-2199-13-22-S4.zip › hkgFinder/hkglist.html]

 R output 


import namespace="mml" implementation="#mathplayer"?


# List of Potential Housekeeping Genes

 

NULL

| |  | gene | SD | logFoldChange | FoldChange | p | p.adj | | --- | --- | --- | --- | --- | --- | --- | | 3 | Cg18S rRNA | 0.19 | -0.35 | 1.3 | 0.000166917 | 0.0011 | | 5 | Cg25S rRNA | 0.22 | -0.39 | 1.3 | 0.000043139 | 0.0004 | | 1 | Cg5.8S rRNA | 0.26 | -0.46 | 1.4 | 0.011029945 | 0.0154 | | 15 | CgPGK1 | 0.87 | -1.56 | 3.0 | 0.007705210 | 0.0154 | | 13 | CgGAPDH | 1.19 | -2.18 | 4.5 | 0.000003005 | 0.0000 | | 6 | 84U CgCDR1 | 1.36 | -2.49 | 5.6 | 0.000025397 | 0.0003 | | 8 | 84U CgPdr1 | 1.40 | -2.56 | 5.9 | 0.000000014 | 0.0000 | | 11 | CgEF1a | 1.58 | -2.89 | 7.4 | 0.000019649 | 0.0002 | | 16 | 84U CgERG11 | 1.66 | -3.02 | 8.1 | 0.000555393 | 0.0028 | | 9 | CgCyclophilin | 1.71 | -3.12 | 8.7 | 0.000000339 | 0.0000 | | 2 | CgRPL2A | 2.01 | -3.67 | 12.7 | 0.000575069 | 0.0028 | | 12 | 84U CgERG4 | 2.16 | -3.93 | 15.3 | 0.000087806 | 0.0007 | | 10 | 84U CgERG2 | 2.21 | -4.02 | 16.3 | 0.000162493 | 0.0011 | | 4 | CgRPL10 | 2.26 | -4.12 | 17.4 | 0.000000654 | 0.0000 | | 7 | 84U CgACT1 | 2.44 | -4.45 | 21.8 | 0.000635370 | 0.0028 | | 14 | 84U CgERG10 | 2.58 | -4.72 | 26.3 | 0.000000074 | 0.0000 | |

  
 

NULL

Genes with the smallest SD and smallest FoldChange make the best housekeeping genes.

 

NULL

---


Generated on: *Thu Dec 22 13:53:51 2011* - **R2HTML**


---
