# Supplementary material for: Evaluation of reference genes for real-time quantitative PCR studies in Candida glabrata following azole treatment
Source: BMC Mol Biol. 2012 Jun 29;13:22. doi: 10.1186/1471-2199-13-22 (PMC3482582; doi:10.1186/1471-2199-13-22)
Supplement: Additional file 4 — hkgFinder. [file 1471-2199-13-22-S4.zip › hkgFinder/reslist.html]

 R output 


import namespace="mml" implementation="#mathplayer"?


# Final Statistical Results

| |  | gene | ddCt | FoldChange | p | p.adj | | --- | --- | --- | --- | --- | --- | | 7 | 84U CgPdr1 | -2.210 | 4.6 | 0.000000023 | 0.0000 | | 13 | 84U CgERG10 | -4.370 | 20.7 | 0.000000101 | 0.0000 | | 8 | CgCyclophilin | -2.777 | 6.9 | 0.000000533 | 0.0000 | | 3 | CgRPL10 | -3.770 | 13.6 | 0.000000900 | 0.0000 | | 12 | CgGAPDH | -1.830 | 3.6 | 0.000005493 | 0.0001 | | 10 | CgEF1a | -2.543 | 5.8 | 0.000026746 | 0.0003 | | 5 | 84U CgCDR1 | -2.143 | 4.4 | 0.000037273 | 0.0003 | | 11 | 84U CgERG4 | -3.587 | 12.0 | 0.000111581 | 0.0009 | | 9 | 84U CgERG2 | -3.677 | 12.8 | 0.000204371 | 0.0014 | | 2 | CgRPL2A | -3.323 | 10.0 | 0.000705221 | 0.0042 | | 6 | 84U CgACT1 | -4.100 | 17.1 | 0.000751010 | 0.0042 | | 15 | 84U CgERG11 | -2.670 | 6.4 | 0.000761995 | 0.0042 | | 14 | CgPGK1 | -1.217 | 2.3 | 0.013113991 | 0.0393 | | 4 | Cg25S rRNA | -0.047 | 1.0 | 0.068902721 | 0.1378 | | 1 | Cg5.8S rRNA | -0.117 | 1.1 | 0.148721809 | 0.1487 | |

  

Script written by Jeff Skinner (contractor with Lockheed Martin) for NIAID OCICB.

---


Generated on: *Mon Dec 05 14:03:14 2011* - **R2HTML**


---
