# Supplementary figures and images for: Evaluation of reference genes for real-time quantitative PCR studies in Candida glabrata following azole treatment
Source: BMC Mol Biol. 2012 Jun 29;13:22. doi: 10.1186/1471-2199-13-22 (PMC3482582; doi:10.1186/1471-2199-13-22)

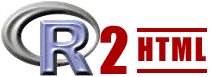

Supplement: Additional file 4 — hkgFinder. [file 1471-2199-13-22-S4.zip › hkgFinder/R2HTMLlogo.gif]
